# Computational Valuation of Darcy Ternary-Hybrid Nanofluid Flow across an Extending Cylinder with Induction Effects

**DOI:** 10.3390/mi13040588

**Published:** 2022-04-09

**Authors:** Khalid Abdulkhaliq M. Alharbi, Ahmed El-Sayed Ahmed, Maawiya Ould Sidi, Nandalur Ameer Ahammad, Abdullah Mohamed, Mohammed A. El-Shorbagy, Muhammad Bilal, Riadh Marzouki

**Affiliations:** 1 Mechanical Engineering Department, College of Engineering, Umm Al-Qura University, Makkah 24382, Saudi Arabia; kamharbi@uqu.edu.sa; 2 Mathematics Department, Faculty of Science, Taif University, Taif 21944, Saudi Arabia; a.elsayed@tu.edu.sa; 3 RT-M2A Laboratory, Mathematics Department, College of Science, Jouf University, Sakaka 72311, Saudi Arabia; msidi@ju.edu.sa; 4 Computational & Analytical Mathematics and Their Applications Research Group, Department of Mathematics, Faculty of Science, University of Tabuk, Tabuk 71491, Saudi Arabia; n.ameer1234@gmail.com; 5 Research Centre, Future University in Egypt, New Cairo 11745, Egypt; mohamed.a@fue.edu.eg; 6 Department of Mathematics, College of Science and Humanities in Al-Kharj, Prince Sattam bin Abdulaziz University, Al-Kharj 11942, Saudi Arabia; ma.hassan@psau.edu.sa; 7 Department of Basic Engineering Science, Faculty of Engineering, Menoufia University, Shebin El-Kom 32511, Egypt; 8 Department of Mathematics, City University of Science and Information Technology, Peshawar 25000, Pakistan; 9 Chemistry Department, College of Science, King Khalid University, Abha 61413, Saudi Arabia; rmarzouki@kku.edu.sa

**Keywords:** electromagnetic induction, ternary hybrid nanofluid, stretching cylinder, Parametric Continuation Method (PCM), chemical reaction, heat source

## Abstract

The flow of an electroconductive incompressible ternary hybrid nanofluid with heat conduction in a boundary layer including metallic nanoparticles (NPs) over an extended cylindrical with magnetic induction effects is reported in this research. The ternary hybrid nanofluid has been synthesized with the dispersion of titanium dioxide, cobalt ferrite, and magnesium oxide NPs in the base fluid water. For a range of economical and biological applications, a computational model is designed to augment the mass and energy conveyance rate and promote the performance and efficiency of thermal energy propagation. The model has been written as a system of partial differential equations. Which are simplified to the system of ordinary differential equations through similarity replacements. The computing approach parametric continuation method is used to further process the resultant first order differential equations. The results are validated with the bvp4c package for accuracy and validity. The outcomes are displayed and analyzed through Figures and Tables. It has been observed that the inverse Prandtl magnetic number and a larger magnetic constant reduce the fluid flow and elevate the energy profile. The variation of ternary hybrid NPs significantly boosts the thermophysical features of the base fluid.

## 1. Introduction

In general, vortex shedding around a circular cylinder cause resonance and structural vibrations, as well as increases in mean drag and lift fluctuations. As a result, vortex shedding flow management is important for engineering applications, as it can successfully result in vibration and noise reduction, drag reduction, and flow separation suppression [1]. Flow control over a circular cylinder has a long history of research. Many engineering methods use flow inside a cylinder, but flow over a canister in a limited domain, such as a stream in a lateral trench or pipe flow, has received significantly less attention. Many situations, such as blood flow across veins from operating sources or flow across cylindrical goods near walls, need wall effects to be taken into account while scaling a problem. Furthermore, while natural forms of unorganized and random exterior roughness have been examined, other types of ordered roughness have not. Vibrations during the printing process can cause curliness on the surface of a 3D-printed solid with regular periodic grooves [2]. Salahuddin et al. [3] examined the differently formed nanostructures that affected the mechanical performance and flow efficiency of flow due to rigid and sinusoidal barriers when heat is generated. Wu et al. [4] employed a wind tunnel with multiple active mechanisms to investigate the aerodynamic demands of a 5:1 rectangular sinusoidal radius cylinder. Varying the frequency and amplitude produces a consistent vorticity loop. Bilal et al. [5] studied a non-uniform Maxwell ferrofluid flow through a stretched cylinder with a non-fluctuating suction/injection. When the thermophoresis ratio is increased, the angular momentum of mass propagation increases significantly, while the radial and angular velocity decrease as the viscosity element improves. A statistical assessment of a 3D flow via a rectangular enclosure was demonstrated by Seo et al. [6]. The sinusoidal cylinder was compared against a circular cylinder to see if it could improve total convective efficiency. The cylinder shape had a significant impact on heat transfer, with performance improving by up to 27%. Bilal et al. [7] investigated the CNTs and iron oxide Fe3O4 hybrid nanofluid using an inclined extensible cylinder. According to the research, hybrid NF is the most efficient heat enhancer and may be utilized for both heat transmission and cooling. Refs. [8,9,10] provide some other application uses and flow models.

Ternary hybrid nanofluid is an innovative type of fluid that outperforms at energy exchanges when compared to ordinary fluids, nanofluid, hybrid nanofluid, gasoline, and acetone. Hybrid nanofluids have a wide range of thermal implications, including freezing in high-temperature conditions [11]. Solar energy, heat pumps, heat exchangers, air purifiers, the auto sector, electrical chiller, turbines, nuclear networks, broadcasters, ships, and biotechnology are all examples of hybrid NFs in use. In this work, we are using ternary NPs consisting of titanium dioxide (TiO_2_) cobalt ferrite (CoFe_2_O_4_) and magnesium oxide (MgO). TiO_2_ is a white inorganic compound that has been used in a variety of products for more than a decade. So it is dependable due to non-toxic, phosphorescence, and non-reactive properties, which boost the brilliance and luminosity of a wide range of products while posing no risk of injury. It is the world’s lightest and whitest material, with reflective capabilities, UV absorption and emission qualities, and the ability to protect against skin cancer [12]. Cobalt (Co) and iron (Fe) are metals. Fe lowers interstitial resistance, allowing for charge/ion mobility on the surface and a considerable increase in specific capacitance [13,14]. MgO, often known as magnesia, is a highly absorbent white solid substance that exists organically as reaction products and is a magnesium resource. It is made up of a combination of Mg^2+^ ions and O^2−^ ions bound by electrostatic interactions and has the general structure MgO [15]. The implications of electromagnetic fluxes on the heat transport of water-based iron oxide and CNTs hybrid NFs across dual rotational plates were studied by Bilal et al. [16]. The velocity profile is boosted by the electric variable, while the thermal factor is minimized. Ramesh et al. [17] used Fe_3_O_4_ and CoFe_2_O_4_ in water + EG to study the activation energy features on the movement of hybrid NF across a channel. Wang et al. [18] used a Fe_3_O_4_-MWCNT hybrid NF flow to simulate the impacts of conductive surface and nanocomposites on the thermal conductivity of a conventional solid heat sink. Arif et al. [19] observed ternary hybrid NF with distinct shapes, Al_2_O_3_, Graphene, and CNT. According to the findings, the ternary hybrid nanofluid improves heat transfer rates by up to 33.67% indicating good thermal performance in thermal efficiency. Sahoo et al. [20] addressed the heat transmission of a condenser with a new coolant made up of water-based nanoparticles, such as CNT, Al_2_O_3_, and graphene ternary hybrid NF. Variation in the quantity of NPs from 1 to 3% at 10 L/min resulted in increases of 22.34 and 6.63% in energy exchange, respectively. Fattahi and Karimi [21] conducted thermo-hydrodynamic tests on solar panels with water repellent coatings while employing a ternary hybrid nanofluid. Refs. [22,23,24,25] contain some relevant literature and applications of hybrid NPs in water for biomedical and engineering objectives.

Magnetization is one of the most important aspects in manufacturing and engineering, having a wide range of applications. The interaction of fluid nanoparticles with magnetic fields has an impact on the quality of heat transfer, clutches, and compressors, among other industrial commodities. Magnetic fields have the ability to control and make available the cooling rate of a variety of industrial devices. Interplanetary and astronomy magnetosphere uses, as well as aeronautics and chemical science, all rely on magnetic fields. The flow properties are influenced by the strength and dispersion of the administered metamaterials. Many academics submitted fluid mechanics research publications that described flow characteristics under the influence of a magnetic field. The influence of thermal expansion and a produced magnetic field on the oscillatory conveyance of a fourth order fluid via a vertical tunnel was investigated by Hayat et al. [26]. Raju et al. [27] investigated the effects of heat exchange and the pre-exponential component on MHD free convective flow across a semi plate. Refs. [28,29,30] contain some recent literature on MHD hybrid nanofluid.

To the best of the authors’ knowledge, heat and mass transfer pass over a moving cylinder containing a ternary NPs, as well as magnetic induction, has not been studied to date. As a result, the current research focuses on this topic. In this scenario, the horizontally expanding cylinder is analyzed with a static applied magnetic field. With adequate far stream and wall conditions, the dynamic model is transformed into a system of ODEs. To obtain the solutions, the computational parametric approach process (PCM) is used. The second goal is to increase the efficiency and profitability of thermal energy propagation in a range of economic and biomedical fields.

### Governing Equations

**Figure 1:**
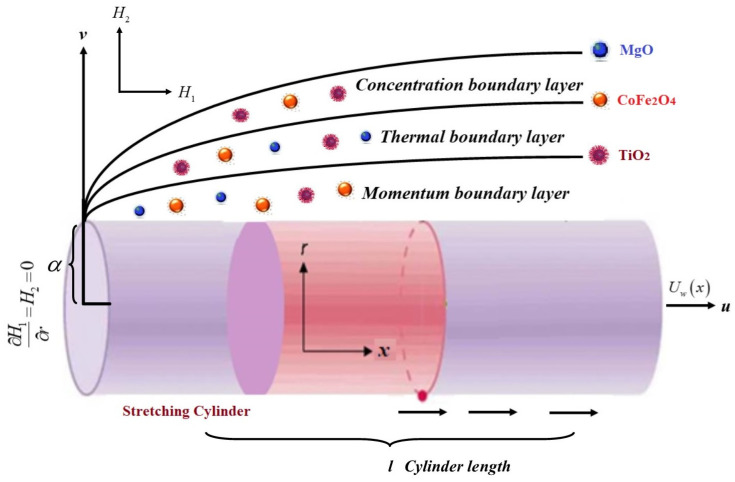
The ternary hybrid nanofluid over a stretching cylinder.

We considered an incompressible axisymmetric trihybrid nanofluid flow over a moving boundary layer cylinder. The *x*-axis is taken along the horizontal cylinder axis, whereas the *r*-axis is supposed towards the radial direction. The effect of induced magnetic is momentous due to the massive Reynolds number, which is consequently distorted by the magnetic effect. The magnetic field is normally applied to mutual constraints, $\bar{H}\left(H_{1},\;\;H_{2}\right)$. The *H*_2_ (parallel component) is the induced magnetic field of the vertical element, which dispels at the surface of the cylinder. *H*_1_ assumed value is $H_{e}=H_{0}x$ at the free stream. The wall and far stream temperature are nominated as $T_{w}$ and $T_{\infty }$. Furthermore, the cylinder is assumed to be extended, with stretching velocity $U_{w}=U_{0}\left(x/l\right),$ towards the axial direction, where $U_{0}$ is constant and *l* is the length of the cylinder, as shown in Figure 1. The fundamental calculations that regulate the fluid flow are defined as [31]:

(1) $$\frac{\partial \left(ru\right)}{\partial x}+\frac{\partial \left(rv\right)}{\partial r}=0,$$

(2) $$\frac{\partial \left(H_{1}r\right)}{\partial x}+\frac{\partial \left(H_{2}r\right)}{\partial r}=0,$$

(3) $$u\frac{\partial u}{\partial x}+v\frac{\partial u}{\partial r}-\frac{\mu_{e}}{4\pi \rho_{Thnf}}\left(H_{1}\frac{\partial H_{1}}{\partial x}+H_{2}\frac{\partial H_{2}}{\partial r}\right)=\frac{\mu_{Thnf}}{\rho_{Thnf}}\left(\frac{\partial^{2}u}{\partial r^{2}}+\frac{1}{r}\frac{\partial u}{\partial r}\right)-\frac{\nu }{k^{*}}u-Fu^{2},$$

(4) $$u\frac{\partial H_{1}}{\partial x}+v\frac{\partial H_{1}}{\partial r}-H_{1}\frac{\partial u}{\partial x}-H_{2}\frac{\partial u}{\partial x}=\eta_{0}\left(\frac{\partial^{2}H_{1}}{\partial r^{2}}+\frac{1}{r}\frac{\partial H_{1}}{\partial r}\right),\;$$

(5) $$u\frac{\partial T}{\partial x}+v\frac{\partial T}{\partial r}=\alpha_{hnf}\left(\frac{\partial^{2}T}{\partial r^{2}}+\frac{1}{r}\frac{\partial T}{\partial r}\right)+\frac{Q_{0}\left(T-T_{\infty }\right)}{\rho C_{p}},\;$$

(6) $$u\frac{\partial C}{\partial x}+v\frac{\partial C}{\partial r}=D_{hnf}\left(\frac{\partial^{2}C}{\partial r^{2}}+\frac{1}{r}\frac{\partial C}{\partial r}\right)-Kc\left(C-C_{\infty }\right),\;$$

Here, Equation (1) denotes the law of mass conservation, which states that mass leaves and enters a system at the same rate. Magnetic flux is expressed mathematically as Equation (2), which provides information about the strength of magnetic flux in a given area of a surface. The component form of the momentum equation that characterizes the behavior of fluid flow is represented in Equation (3). Magnetic induction is represented by Equation (4) as a component form. Equations (5) and (6) are mathematical representations of the heat equation and concentration, respectively, which show the distribution of temperature (thermal energy) and mass around and near the stretching cylinder’s surface, where *Kc* is the rate of chemical reaction, *Q*_0_ is the heat source term, k* is the porosity term, and F is the non-uniform inertia factor constant.

The boundary conditions are:

(7) $$\begin{array}{l} u=U_{w},\;\;\;v=0,\;\;\;\frac{\partial H_{1}}{\partial r}=H_{2}=0,\;\;\;T=T_{w},\;\;\;C=C_{w}\;\;\;\;at\;\;\;\;r=a\\ u\to 0,\;\;v\to 0,\;\;H_{1}\to H_{e}=0,\;\;T\to T_{\infty },\;\;C\to C_{\infty }\;\;\;at\;\;\;\;r\to \infty . \end{array}\}$$

The viscosity model for ternary hybrid nanofluid [32,33]:

(8) $$\frac{\mu_{Thnf}}{\mu_{f}}=\frac{1}{{\left(1-\phi_{\mathrm{MgO}}\right)}^{2.5}{\left(1-\phi_{\mathrm{Ti}O_{2}}\right)}^{2.5}{\left(1-\phi_{\mathrm{Co}Fe_{2}O_{4}}\right)}^{2.5}},$$

The density model for ternary hybrid nanofluid:

(9) $$\frac{\rho_{Thnf}}{\rho_{f}}=\left(1-\phi_{\mathrm{Ti}O_{2}}\right)\left[\left(1-\phi_{\mathrm{Ti}O_{2}}\right)\left\{\left(1-\phi_{CoFe_{2}O_{4}}\right)+\phi_{CoFe_{2}O_{4}}\frac{\rho_{CoFe_{2}O_{4}}}{\rho_{f}}\right\}+\phi_{\mathrm{Ti}O_{2}}\frac{\rho_{\mathrm{Ti}O_{2}}}{\rho_{f}}\right]+\phi_{\mathrm{MgO}}\frac{\rho_{\mathrm{MgO}}}{\rho_{f}},$$

The specific heat model for ternary hybrid nanofluid:

(10) $$\frac{{\left(\rho cp\right)}_{Thnf}}{{\left(\rho cp\right)}_{f}}=\phi_{\mathrm{MgO}}\frac{{\left(\rho cp\right)}_{\mathrm{MgO}}}{{\left(\rho cp\right)}_{f}}+\left(1-\phi_{\mathrm{MgO}}\right)\left[\begin{array}{l} \left(1-\phi_{\mathrm{Ti}O_{2}}\right)\left\{\left(1-\phi_{CoFe_{2}O_{4}}\right)+\phi_{CoFe_{2}O_{4}}\frac{{\left(\rho cp\right)}_{CoFe_{2}O_{4}}}{{\left(\rho cp\right)}_{f}}\right\}\\ +\phi_{\mathrm{Ti}O_{2}}\frac{{\left(\rho cp\right)}_{\mathrm{Ti}O_{2}}}{{\left(\rho cp\right)}_{f}} \end{array}\right]\}$$

The thermal conduction model for ternary hybrid nanofluid:

(11) $$\begin{array}{l} \frac{k_{Thnf}}{k_{hnf}}=\left(\frac{k_{CoFe_{2}O_{4}}+2k_{hnf}-2\phi_{CoFe_{2}O_{4}}\left(k_{hnf}-k_{CoFe_{2}O_{4}}\right)}{k_{CoFe_{2}O_{4}}+2k_{hnf}+\phi_{CoFe_{2}O_{4}}\left(k_{hnf}-k_{CoFe_{2}O_{4}}\right)}\right),\frac{k_{hnf}}{k_{nf}}=\left(\frac{k_{\mathrm{Ti}O_{2}}+2k_{nf}-2\phi_{\mathrm{Ti}O_{2}}\left(k_{nf}-k_{\mathrm{Ti}O_{2}}\right)}{k_{\mathrm{Ti}O_{2}}+2k_{nf}+\phi_{\mathrm{Ti}O_{2}}\left(k_{nf}-k_{\mathrm{Ti}O_{2}}\right)}\right),\\ \frac{k_{nf}}{k_{f}}=\left(\frac{k_{\mathrm{MgO}}+2k_{f}-2\phi_{\mathrm{MgO}}\left(k_{f}-k_{\mathrm{MgO}}\right)}{k_{\mathrm{MgO}}+2k_{f}+\phi_{\mathrm{MgO}}\left(k_{f}-k_{\mathrm{MgO}}\right)}\right), \end{array}\}$$

The electrical conductivity model for ternary hybrid nanofluid [32,33]:

(12) $$\frac{\sigma_{Thnf}}{\sigma_{hnf}}=\begin{array}{l} \left[1+\frac{3\left(\frac{\sigma_{CoFe_{2}O_{4}}}{\sigma_{hnf}}-1\right)\phi_{CoFe_{2}O_{4}}}{\left(\frac{\sigma_{CoFe_{2}O_{4}}}{\sigma_{hnf}}+2\right)-\left(\frac{\sigma_{CoFe_{2}O_{4}}}{\sigma_{hnf}}-1\right)\phi_{CoFe_{2}O_{4}}}\right],\;\frac{\sigma_{hnf}}{\sigma_{nf}}=\left[1+\frac{3\left(\frac{\sigma_{\mathrm{Ti}O_{2}}}{\sigma_{nf}}-1\right)\phi_{\mathrm{Ti}O_{2}}}{\left(\frac{\sigma_{\mathrm{Ti}O_{2}}}{\sigma_{nf}}+2\right)-\left(\frac{\sigma_{\mathrm{Ti}O_{2}}}{\sigma_{nf}}-1\right)\phi_{\mathrm{Ti}O_{2}}}\right],\\ \frac{\sigma_{nf}}{\sigma_{f}}=\left[1+\frac{3\left(\frac{\sigma_{\mathrm{MgO}}}{\sigma_{f}}-1\right)\phi_{\mathrm{MgO}}}{\left(\frac{\sigma_{\mathrm{MgO}}}{\sigma_{f}}+2\right)-\left(\frac{\sigma_{\mathrm{MgO}}}{\sigma_{f}}-1\right)\phi_{\mathrm{MgO}}}\right] \end{array}\}$$

The transformation variables used in Equations (1)–(6) are as follows:

(13) $$\begin{array}{l} \psi \left(x,\;r\right)={\left(\nu_{f}U_{w}x\right)}^{\frac{1}{2}}a\;f\left(\eta \right),\;\;\;\eta \left(x,\;r\right)=\frac{r^{2}-a^{2}}{2a}{\left(\frac{U_{w}}{\nu_{f}x}\right)}^{\frac{1}{2}},\;\;\;H_{1}=rH_{0}g\left(\eta \right),\;\;\;\;T_{w}=T_{\infty }+T_{0}\left(x/l\right),\\ \theta \left(\eta \right)=\frac{T-T_{\infty }}{T_{w}-T_{\infty }},\;\;\;\varphi \left(\eta \right)=\frac{C-C_{\infty }}{C_{w}-C_{\infty }}. \end{array}\}$$

For momentum equations, the similarity variables are:

$$u=\frac{1}{r}\frac{\partial \psi^{*}}{\partial r},\;\;\;\;v=-\frac{1}{r}\frac{\partial \psi^{*}}{\partial x}.$$

By incorporating Equation (13), we find:

(14) $$\left(1+2\gamma \eta \right)f^{\prime \prime \prime }+2\gamma f^{\prime \prime }+\vartheta_{1}\vartheta_{2}\left(ff^{\prime \prime }-Fr{f^{\prime }}^{2}+\beta \left({g^{\prime }}^{2}-gg^{\prime \prime }\right)\right)=0,$$

(15) $$\lambda \left(1+2\gamma \eta \right)g^{\prime \prime }+2\gamma \lambda g^{\prime \prime }+\left(fg^{\prime \prime }-f^{\prime \prime }g\right)=0,\;$$

(16) $$\vartheta_{4}\left(\left(1+2\gamma \eta \right)\theta^{\prime \prime }+2\gamma \theta^{\prime }\right)+\vartheta_{3}Pr\left(f\theta^{\prime }-f^{\prime }\theta \right)+Q_{1}\theta =0.\;$$

(17) $$\left(\left(1+2\gamma \eta \right)\varphi^{\prime \prime }+2\gamma \varphi^{\prime }\right)+\vartheta_{5}Sc\left(f\varphi^{\prime }-f^{\prime }Kr\right)=0.$$

Here,

$$\vartheta_{1}=\frac{\mu_{Thnf}}{\mu_{bf}},\vartheta_{2}=\frac{\rho_{Thnf}}{\rho_{bf}},\vartheta_{3}=\frac{{\left(\rho C_{p}\right)}_{Thnf}}{{\left(\rho C_{p}\right)}_{bf}},\vartheta_{4}=\frac{k_{Thnf}}{k_{bf}},\vartheta_{5}=\frac{D_{Thnf}}{D_{bf}}.\;$$

The transform conditions are:

(18) $$\begin{array}{l} f\left(0\right)=0,\;\;f^{\prime }\left(0\right)=1,\;\;g^{\prime }\left(0\right)=0,\;\;g\left(0\right)=0,\;\;\theta \left(0\right)=1,\;\;\varphi \left(0\right)=1\;\;\;\;\mathrm{when}\;\;\;\eta =0\\ f^{\prime }\left(\infty \right)\to 0,\;\;g^{\prime }\left(\infty \right)\to 1,\;\;\theta \left(\infty \right)\to 0,\;\;\varphi \left(\infty \right)\to 0\;\;\;\;as\;\;\;\eta \to 0 \end{array}$$

Here, β is the magnetic parameters, *γ* is the curvature term, λ the inverse of magnetic Prandtl number, *Pr* is the Prandtl number, *Fr* Forchheimer number, $Q_{1}$ is the heat absorption and generation term, *Sc* is the Schmidt number, and *Kr* is the rate of chemical reaction defined as:

(19) $$\begin{array}{l} \beta =\frac{\mu_{e}}{4\pi \rho_{f}}{\left(\frac{H_{0}l}{U_{0}}\right)}^{2},\;\;\;\gamma ={\left(\frac{\nu_{f}l}{U_{0}a^{2}}\right)}^{\frac{1}{2}},\;\;\lambda =\frac{\eta_{0}}{\nu_{f}},\;\;\;Pr=\frac{\mu_{f}}{\rho_{f}}\frac{\left(\rho C_{p}\right)}{k_{f}},\;\;\;Fr=\frac{C_{b}}{k^{*1/2}}x,\\ Q_{1}=\frac{xQ_{0}}{\rho C_{p}},\;\;\;Sc=\frac{\upsilon_{f}}{D_{f}},\;\;\;Kr=\frac{Kc\;l}{U_{0}}. \end{array}\}$$

The dragging friction, wall thermal gradient, and Sherwood number are [32]:

(20) $$C_{f_{x}}=\frac{\tau_{w}}{u_{w}^{2}\rho_{f}},\;\;\;Nu=\frac{q_{w}\;x}{\left(T_{w}-T_{\infty }\right)k_{f}},\;\;\;\;Sh=\frac{j_{w}\;x}{\left(C_{w}-C_{\infty }\right)D_{f}}.$$

where

(21) $$\tau_{w}=\mu_{hnf}{\left(\frac{\partial u}{\partial r}\right)}_{r=a},\;\;\;\;q_{w}=-k_{hnf}{\left(\frac{dT}{dr}\right)}_{r=a},\;\;\;j_{w}=-D_{hnf}{\left(\frac{dC}{dr}\right)}_{r=a}.$$

The dimensionless form of Equation (20) is:

(22) $$Re^{\frac{1}{2}}C_{f}=\frac{{\left(1-\phi_{2}\right)}^{2.5}}{{\left(1-\phi_{1}\right)}^{2.5}{\left(1-\phi_{3}\right)}^{2.5}}f^{\prime \prime }\left(0\right),\;\;\;\;Re^{-\frac{1}{2}}Nu_{x}=-\frac{k_{hnf}}{k_{f}}\theta^{\prime }(0),\;\;Re^{\frac{-1}{2}}Sh=-\varphi^{\prime }(0).$$

Here,

$$\phi_{1}=\phi_{\mathrm{Ti}O_{2}},\;\;\;\phi_{2}=\phi_{\mathrm{Co}Fe_{2}O_{4}},\;\;\;\phi_{3}=\phi_{\mathrm{MgO}}.$$

## 2. Solution Procedures

The subsequent stages demonstrate the fundamental steps of applying the PCM approach to a system of ODEs (14)–(17) with a boundary condition (18) [34,35,36,37]:

**Step 1: Reducing the BVP to a first-order system ODEs**

(23) $$\begin{array}{l} \hbar_{1}=f(\eta ),\;\;\;\hbar_{2}=f^{\prime }(\eta ),\;\;\;\hbar_{3}=f^{\prime \prime }(\eta ),\;\;\;\hbar_{4}=g(\eta ),\;\;\;\hbar_{5}=g^{\prime }(\eta ),\\ \hbar_{6}=\theta (\eta ),\;\;\;\hbar_{7}=\theta^{\prime }(\eta ),\;\;\;\;\hbar_{8}=\varphi (\eta ),\;\;\;\;\hbar_{9}(\eta )=\varphi^{\prime }(\eta ). \end{array}\}$$

By putting (22) in (11)–(16) and (17), we find:

(24) $$\left(1+2\gamma \eta \right){\hbar^{\prime }}_{3}+2\gamma \hbar_{3}+\vartheta_{2}\left(\hbar_{1}\hbar_{3}-Fr\hbar_{2}{}^{2}+\beta \left(\hbar_{5}{}^{2}-\hbar_{4}{\hbar^{\prime }}_{5}\right)\right)=0,$$

(25) $$\lambda \left(1+2\gamma \eta \right){\hbar^{\prime }}_{5}+2\gamma \lambda {\hbar^{\prime }}_{5}+\left(\hbar_{1}{\hbar^{\prime }}_{5}-{\hbar^{\prime }}_{3}\hbar_{4}\right)=0,\;$$

(26) $$\vartheta_{4}\left(\left(1+2\gamma \eta \right){\hbar^{\prime }}_{7}+2\gamma \hbar_{7}\right)+\vartheta_{3}Pr\left(\hbar_{1}\hbar_{7}-\hbar_{2}\hbar_{6}\right)+Q_{1}\hbar_{6}=0.\;$$

(27) $$\left(\left(1+2\gamma \eta \right){\hbar^{\prime }}_{9}+2\gamma \hbar_{9}\right)+\vartheta_{5}Sc\left(\hbar_{1}\hbar_{9}-\hbar_{2}Kr\right)=0.$$

the transform conditions are:

(28) $$\begin{array}{l} \hbar_{1}\left(0\right)=0,\;\;\hbar_{2}\left(0\right)=1,\;\;\hbar_{15}\left(0\right)=0,\;\;\hbar_{4}\left(0\right)=0,\;\;\hbar_{6}\left(0\right)=1,\;\;\hbar_{8}\left(0\right)=1\;\;\;\;\mathrm{when}\;\;\;\eta =0\\ \hbar_{2}\left(\infty \right)\to 0,\;\;\hbar_{15}\left(\infty \right)\to 1,\;\;\hbar_{6}\left(\infty \right)\to 0,\;\;\hbar_{8}\left(\infty \right)\to 0\;\;\;\;as\;\;\;\eta \to 0 \end{array}$$

**Step 2: Introducing parameter *p***

(29) $$\left(1+2\gamma \eta \right){\hbar^{\prime }}_{3}+2\gamma \hbar_{3}+\vartheta_{2}\left(\hbar_{1}\left(\hbar_{3}-1\right)p-Fr\hbar_{2}{}^{2}+\beta \left(\hbar_{5}{}^{2}-\hbar_{4}{\hbar^{\prime }}_{5}\right)\right)=0,$$

(30) $$\lambda \left(1+2\gamma \eta \right){\hbar^{\prime }}_{5}+2\gamma \lambda \left(\hbar_{5}-1\right)p+\left(\hbar_{1}{\hbar^{\prime }}_{5}-{\hbar^{\prime }}_{3}\hbar_{4}\right)=0,\;$$

(31) $$\vartheta_{4}\left(\left(1+2\gamma \eta \right){\hbar^{\prime }}_{7}+2\gamma \left(\hbar_{7}-1\right)p\right)+\vartheta_{3}Pr\left(\hbar_{1}\hbar_{7}-\hbar_{2}\hbar_{6}\right)+Q_{1}\hbar_{6}=0.\;$$

(32) $$\left(\left(1+2\gamma \eta \right){\hbar^{\prime }}_{9}+2\gamma \left(\hbar_{9}-1\right)p\right)+\vartheta_{5}Sc\left(\hbar_{1}\hbar_{9}-\hbar_{2}Kr\right)=0.$$

**Step 3: Differentiating by parameter ‘*p*’**

By differentiating Equations (29)–(32) w.r.t parameter *p*, we find:

(33) $$V^{\prime }=AV+R,$$

and

(34) $$\frac{d\zeta_{i}}{d\tau }$$

where *i* = 1, 2,…,11.

**Step 4: Apply the superposition principle**

(35) V=aU+W,

For each element, we resolve the two Cauchy problems listed below.

(36) U=aU,

(37) $$W^{\prime }=AW+R,$$

By putting Equation (37) in Equation (35), we find

(38) $$(aU+W{)}^{\prime }=A(aU+W)+R,$$

**Step 5: Solving the Cauchy problems**

This study makes use of a numerical implicit system, as seen below.

(39) $$\begin{array}{l} \frac{U^{i+1}-U^{i}}{\Delta \eta }=AU^{i+1},\;\;or\;\;\;\;(I-\Delta \eta A)U^{i+1}=U^{i},\\ \frac{W^{i+1}-W^{i}}{\Delta \eta }=AW^{i+1},\;\;or\;\;\;\;(I-\Delta \eta A)W^{i+1}=W^{i}. \end{array}\}$$

Finally, we see the iterative form as:

(40) $$\begin{array}{l} U^{i+1}={\left(I-\Delta \eta A\right)}^{-1}U^{i},\\ W^{i+1}={\left(I-\Delta \eta A\right)}^{-1}(W^{i}+\Delta \eta R). \end{array}\}$$

## 3. Results and Discussion

The graphical results of the system of DEs are assessed by commissioning the computational methodology “parametric continuation approach”. The following are some of the conclusions that have been perceived:

### 3.1. Velocity Profile $f^{\prime }\left(\eta \right)$

**Figure 2:**
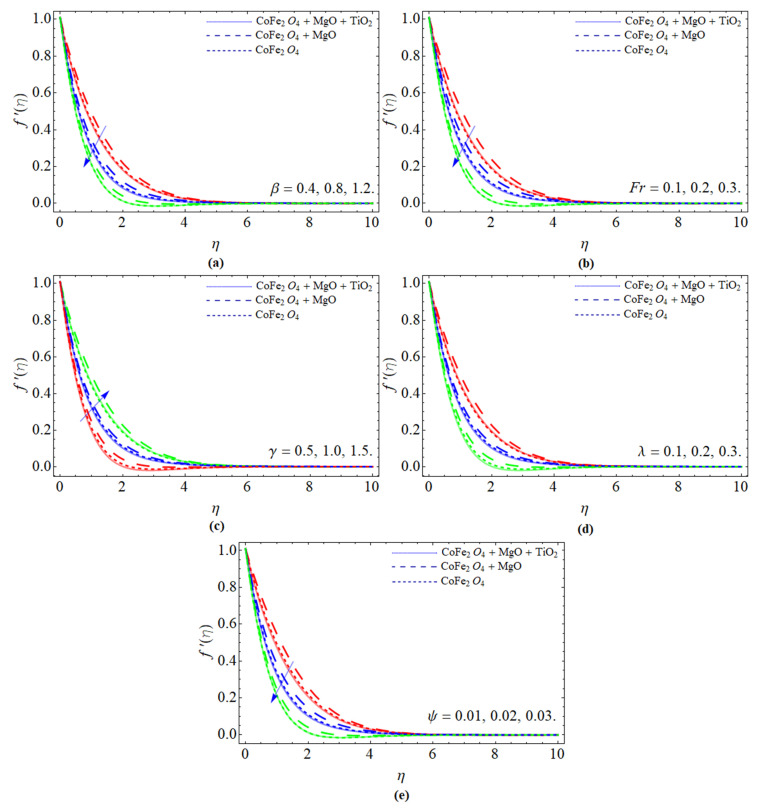
The presentation of velocity $f^{\prime }\left(\eta \right)$ profile against the variation of (**a**) magnetic term β, (**b**) Darcy Forchhemier term *Fr*, (**c**) curvature constant γ, (**d**) inverse magnetic Prandtl number λ, and (**e**) nanoparticles volume friction ψ.

Figure 2a–e highlighted the presentation of velocity $f^{\prime }\left(\eta \right)$ profile against the variation of the magnetic term β, Darcy Forchhemier term *Fr*, Curvature constant γ, Inverse magnetic Prandtl number λ, and nanoparticles volume friction ψ, respectively. In the present analysis the value of ψ is used as $\psi =\phi_{1}=\phi_{2}=\phi_{3}.$ Figure 2a,b exposed that the fluid velocity profile reduces with the effect of magnetic term β and Darcy Forchhemier term *Fr*. Physically, the surface permeability reduces with the rising effect of Darcy Forchhemier number, which results in the reduction in the momentum boundary layer. Therefore, such a scenario has been noticed. Figure 2c,d revealed that the curvature constant γ enhances the velocity profile, while the influence of inverse magnetic Prandtl number λ and nanoparticles volume friction ψ declines the momentum boundary layer, respectively. The curvature variable is lowered as the cylinder radius grows (inverse relation). In contrast, a shorter cylinder radius results in a stronger curvature impact, which promotes momentum propagation and velocity augmentation in the boundary layer as shown in Figure 2c. The rising quantity of nanoparticles enhances the density and visocsity of base fluid, which causes the reduction in the velocity profile (Figure 2e).

### 3.2. Magnetic Induction $g^{\prime }\left(\eta \right)$

**Figure 3:**
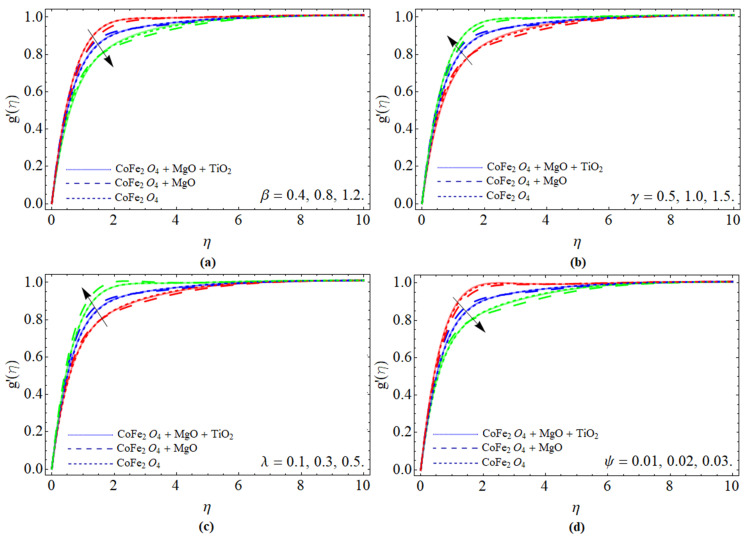
The presentation of induced magnetic field $g^{\prime }\left(\eta \right)$ versus the variation of (**a**) magnetic term β, (**b**) curvature constant γ, (**c**) inverse magnetic Prandtl number λ, and (**d**) nanoparticles volume friction ψ.

Figure 3a–d emphasize the presentation of induced magnetic field $g^{\prime }\left(\eta \right)$ versus the variation of the magnetic term β, curvature constant γ, inverse magnetic Prandtl number λ, and nanoparticles volume friction ψ, respectively. Figure 3a indicated that the magnetic induction profile reduces with the upshot of the magnetic term. The phenomenon is observed due to the opposing effect generated by the Lorentz force. Figure 3b,c display that the magnetic gradient function accelerated with the improving values of curvature constant γ and inverse magnetic Prandtl number. A larger curvature impact boosts the magnetic induction, signifying that a lower surface size of the extending cylinder facilitates magnetic dissemination as highlighted in Figure 3b. Changing the quantity λ creates reinforcement for the stream magnetic gradient curve. In the far flow field, the flow allocation evolves to a unity terminal in the reported cases. The magnetic Prandtl number is the relation of the frequency of magnetic dispersion to the rate of viscous diffusion. The reciprocal ratio, or the rate of viscous dissemination divided by the rate of magnetic diffusion, is denoted by λ. Figure 3d elaborated that the inclusion of ternary nanoparticles diminishes the induced magnetic field and fluid temperature. This is the actual property of ternary NPs, which makes them more efficient for industrial and biomedical uses. The rising quantity of NPs enhances the fluid kinetic viscosity, which results in a reduction in energy and velocity profile as well as magnetic induction $g^{\prime }\left(\eta \right)$.

### 3.3. Temperature Profile θ(η)

**Figure 4:**
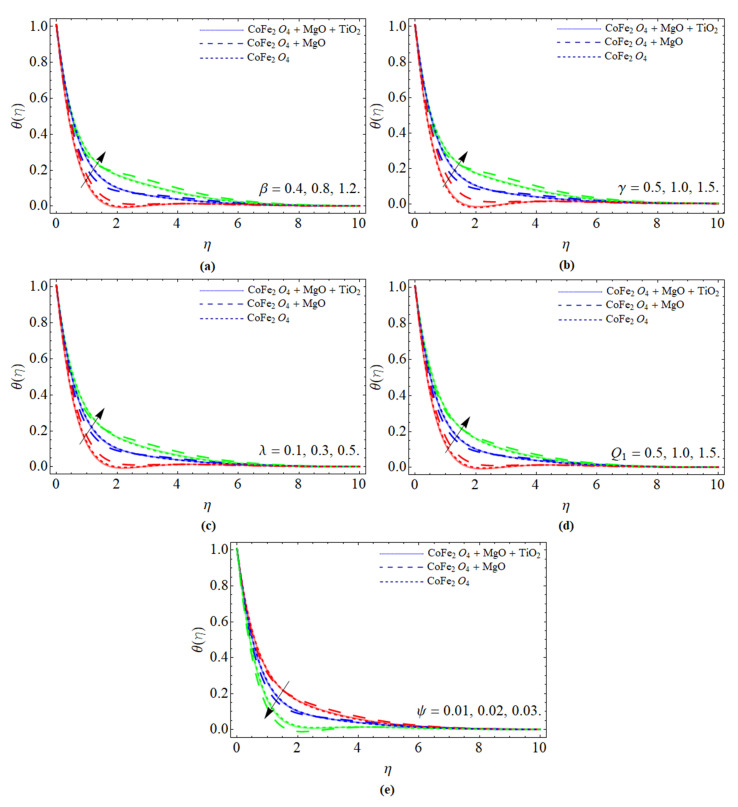
The presentation of energy θ(η) profile against the variation of (**a**) magnetic term β, (**b**) curvature constant γ, (**c**) inverse magnetic Prandtl number λ, (**d**) heat source $Q_{1}$, and (**e**) nanoparticles volume friction ψ.

Figure 4a–d exemplify the presentation of energy θ(η) profile against the variation of the magnetic term β, curvature constant γ, inverse magnetic Prandtl number λ, heat source $Q_{1}$, and nanoparticles volume friction ψ, respectively. Figure 4a,b explain that the energy θ(η) profile boosts with the positive variation of magnetic term β and curvature constant. Physically, the friction produces due to Lorentz, which generates heat energy as well; as a result, the energy profile is enhanced. The variation in inverse magnetic Prandtl number λ and heat source $Q_{1}$ boosts the temperature of the fluid as seen in Figure 4c,d. Magnetic diffusion is consequently more helpful to the thermal diffusion process than viscous (momentum) diffusion, therefore such phenomena have been noticed in Figure 4c. On the other hand, the effect of a heat source is generated, which causes the rise in energy profile shown in Figure 4d. As we have discussed in Figure 2 and Figure 3, the inclusion of ternary nanoparticles improves the energy-absorbing capability of the base fluid, which eventually lowers the temperature θ(η) of the fluid as perceived in Figure 4e.

### 3.4. Concentration Profile φ(η)

**Figure 5:**
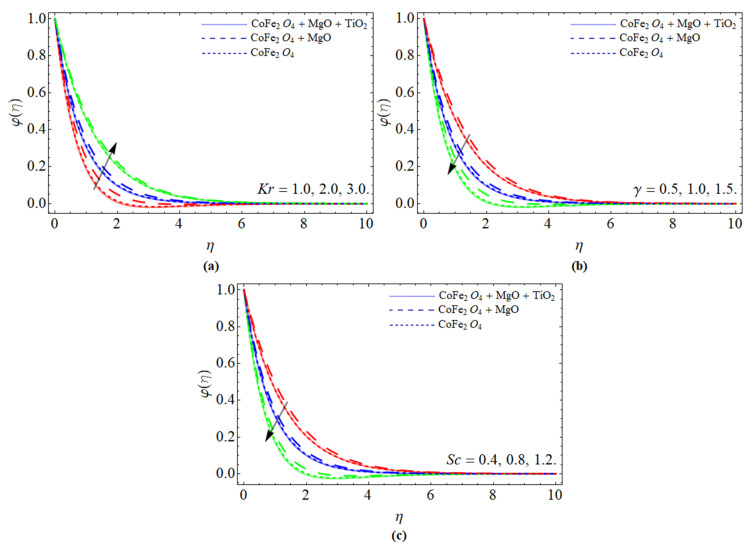
The presentation of concentration φ(η) profile against the variation of (**a**) chemical reaction term *Kr*, (**b**) curvature constant γ, and (**c**) Schmidt number *Sc*.

Figure 5a–c report the presentation of concentration φ(η) profile against the variation of chemical reaction term *Kr*, curvature constant γ, and Schmidt number *Sc*, respectively. The chemical reaction coefficient positively affects the mass transfer, because their effect encourages fluid particles to move fast, which results in the positive variation as elaborated in Figure 5a. Figure 5b,c display that the consequences of curvature constant γ and Schmidt number *Sc* declines the mass transfer profile. The kinetic viscosity of fluid enhances with the influence of Schmidt number, which lowers the mass transfer φ(η) of fluid.

**Figure 6:**
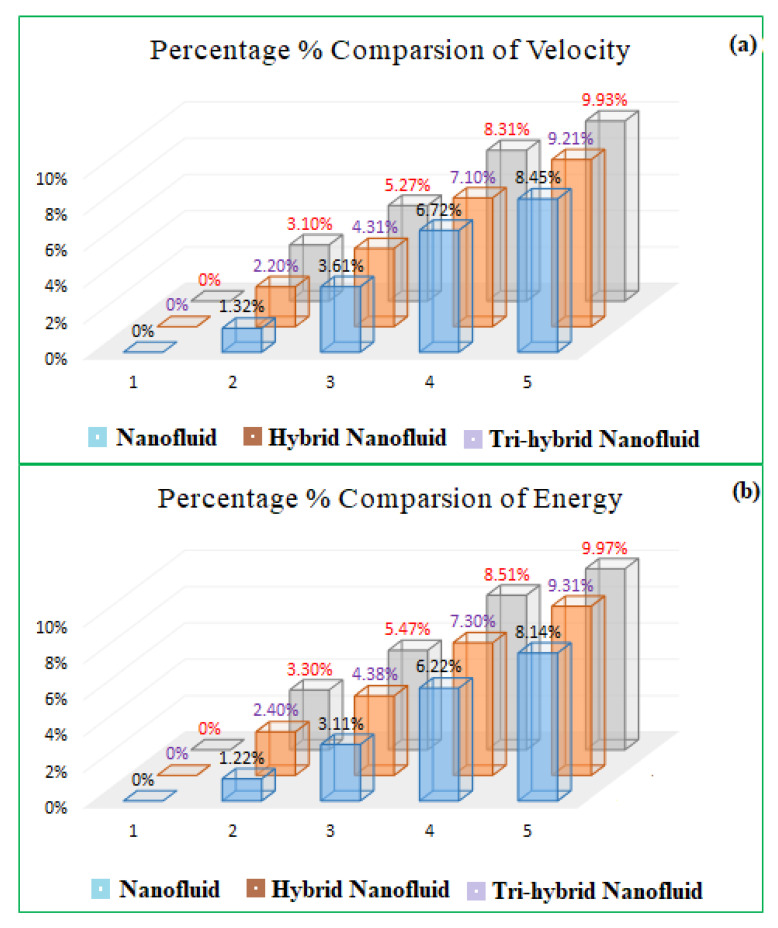
The percentage comparison of nanofluid, hybrid, and ternary hybrid nanofluid of velocity and energy in (**a**,**b**) respectively.

Table 1 illustrated the experimental values and thermochemical properties of the base fluid, magnesium oxide MgO, titanium dioxide TiO_2_ and Cobalt ferrite Fe_2_O_4_, respectively. Table 2 and Table 3 described the statistical valuation of PCM and bvp4c techniques, to confirm the legality of the current report. The energy field and mass transition profile are associated with the purpose. Table 2 and Table 3 also exposed the relative calculations between hybrid (TiO_2_ + Fe_2_O_4_) and ternary hybrid NF (MgO + TiO_2_ + Fe_2_O_4_). It has been professed that the mass and heat allocation percentage of ternary hybrid NF as compared to hybrid nanofluid NF or ordinary fluid is superior. Figure 6a,b emphasized the relative analysis of simple (TiO_2_ or CoFe_2_O_4_ or MgO), hybrid nanofluid (TiO_2_ + MgO/water) and trihybrid nanofluid (CoFe_2_O_4_ + TiO_2_ + MgO/water) for the velocity and energy profile. It has been observed that the tri-hybrid and hybrid nanofluid has a great tendency for the fluid energy and velocity transference rate as compared to the simple NF.

## 4. Conclusions

An incompressible electroconductive ternary hybrid nanofluid flow with heat and mass transfer comprised of metallic nanoparticles over a stretching cylindrical has been reported in this study. A computational model is designed to optimize the mass and energy conveying rate, as well as the efficiency and reliability of thermal energy dissemination, for a variety of economic and biological applications. The obtained first order differential equations are subsequently processed using the computational methodology PCM. The accuracy and validity of the results are checked using the bvp4c package. The core findings from the above observation have been presented below:

- The velocity profile reduces with the effect of magnetic term, Darcy Forchhemier term *Fr,* inverse magnetic Prandtl number, and nanoparticles volume fraction, and enhances with the effect of curvature constant.
- The induced magnetic field $g^{\prime }\left(\eta \right)$ augments with the variation of curvature constant and inverse magnetic Prandtl number, while reducing with the volume fraction of nanoparticles and magnetic term.
- The energy θ(η) profile boosts with the positive variation of magnetic term β and curvature constant.
- The variation in inverse magnetic Prandtl number λ and heat source $Q_{1}$ increases the temperature of the ternary nanofluid.
- The chemical reaction coefficient positively affects the mass transfer, because their effect encourages fluid particles to move fast, while the consequences of curvature constant and Schmidt number *Sc* declines the mass transfer profile.
- The variation of ternary hybrid NPs significantly boosts the thermophysical features of the base fluid.

## Figures and Tables

**Table 1:** The experimental values of Titanium dioxide, Cobalt ferrite, magnesium oxide water [32,33].

Base Fluid and Nanoparticles	$\rho \;\;(kg/m^{3})$	k (W/mK)	Cp (j/kgK)	σ (S/m)	$\beta \times 10^{5}\left(K^{-1}\right)\;$
Pure water H_2_O	997.1	0.613	4179	0.05	21
Titanium dioxide TiO_2_	4250	8.9538	686.2	$2.38\times 10^{6}$	0.9
Cobalt ferrite CoFe_2_O_4_	4907	3.7	700	$5.51\times 10^{9}$	-
Magnesium oxide MgO	3560	45	955	$1.42\times 10^{-3}$	1.26

**Table 2:** Comparative assessment for Nusselt number between PCM and bvp4c package with hybrid and ternary hybrid nanofluid.

			PCM	bvp4c	PCM	bvp4c
γ	$Q_{1}$	ψ	$\frac{k_{hnf}}{k_{nf}}\theta^{\prime }(0)$	$\frac{k_{hnf}}{k_{nf}}\theta^{\prime }(0)$	$\frac{k_{Thnf}}{k_{hnf}}\theta^{\prime }(0)$	$\frac{k_{Thnf}}{k_{hnf}}\theta^{\prime }(0)$
0.1			0.0575533	0.0574437	0.0684540	0.0684441
0.3			0.0455121	0.0454040	0.0566133	0.0566033
0.5			0.0465850	0.0464745	0.0569863	0.0569762
0.7			0.0392105	0.0391005	0.0471417	0.0571333
	0.0		0.0665586	0.0464478	0.0754565	0.0554466
	0.2		0.0675762	0.0474654	0.0775971	0.0575864
	0.4		0.0679960	0.0478863	0.05789975	0.0589856
	0.6	0.01	0.0774418	0.0573431	0.0883470	0.0683360
		0.02	0.0784230	0.0583222	0.0893281	0.0693170
		0.03	0.0791322	0.0590315	0.0813152	0.0713051
		0.04	0.0822417	0.0623409	0.0943329	0.0843227

**Table 3:** Comparative assessment for Sherwood number between PCM and bvp4c package with hybrid and ternary hybrid nanofluid.

		PCM	bvp4c	PCM	bvp4c
*Kr*	φ	$\frac{D_{hnf}}{D_{nf}}\varphi^{\prime }(0)$	$\frac{D_{hnf}}{D_{nf}}\varphi^{\prime }(0)$	$\frac{D_{Thnf}}{D_{hnf}}\varphi^{\prime }(0)$	$\frac{D_{Thnf}}{D_{hnf}}\varphi^{\prime }(0)$
0.3		0.0532438	0.0532227	0.0742420	0.0742210
0.6		0.0529432	0.0529211	0.0739436	0.0739224
0.9		0.0515954	0.0515741	0.0714945	0.0714733
1.2		0.4930352	0.4930161	0.6910340	0.6910131
	0.01	0.1627703	0.1627510	0.2677735	0.2677624
	0.02	0.1638823	0.1638631	0.2728854	0.2728641
	0.03	0.5687541	0.5687341	0.7774604	0.7774412
	0.04	0.6026629	0.6026616	0.8906814	0.8906603

## Data Availability

No data is available for this study.
